# Progesterone Regulates Glucose Metabolism Through Glucose Transporter 1 to Promote Endometrial Receptivity

**DOI:** 10.3389/fphys.2020.543148

**Published:** 2020-09-24

**Authors:** Hongshuo Zhang, Jia Qi, Yufei Wang, Jing Sun, Zhen Li, Linlin Sui, Jianhui Fan, Chao Liu, Yuhong Shang, Li Kong, Ying Kong

**Affiliations:** ^1^Core Laboratory of Glycobiology and Glycoengineering, College of Basic Medical Sciences, Dalian Medical University, Dalian, China; ^2^Department of Ultrasound, Xijing Hospital, Xi’an, China; ^3^Department of Gynecology, First Affiliated Hospital of Dalian Medical University, Dalian, China

**Keywords:** progesterone, glucose metabolism, glucose transporter 1, endometrial receptivity, implantation

## Abstract

Successful embryo implantation requires receptive endometrium, which is conducive to the process of embryo recognition, adhesion, and invasion within a certain period of time and is inseparable from the dynamic interaction between 17β-estradiol (E2) and progesterone (P4). Proper glucose metabolism is critical for the profound physiological changes in the endometrium entering the receptive state. And glucose transporters (GLUTs) are responsible for intracellular uptake of glucose and are the first step in glucose metabolism. Prior literature has reported the presence of GLUTs in the endometrium. However, we still do not understand the specific mechanisms of this process. In this study, we identified the effect of P4 on glucose transporter 1 (GLUT1) using *in vivo* animal models and determined the regulation of glucose metabolism by P4 in cells. We highly suspect that this pregnancy failure may be due to reduced GLUT1-mediated glucose metabolism, resulting in a decrease in endometrial receptivity caused by an inadequate energy supply and synthesis of substrate. Here, we propose a possible mechanism to explain how embryo implantation is affected by P4 and glucose utilization under abnormal endometrial conditions.

## Introduction

Embryo implantation is a key process in the establishment of mammalian pregnancy. The endometrium must be in a receptive state to successfully complete this step. This complex series of events occurs during a restricted time period, known as the “window of implantation” (Teh et al., 2016). Poor receptivity of the uterus during implantation may be the cause of implantation failure in women with repeated failed pregnancies or multiple “*in vitro* fertilization” (IVF) failures. Human pregnancy is less efficient (about 30%), and implantation failure accounts for 75% of pregnancy failure (Norwitz et al., 2001). The root cause of early pregnancy failure may be that the endometrium is not properly differentiated to the receptive state, even when the embryo is developing normally (Carson et al., 2000; Gellersen et al., 2007). At the same time, some studies have shown that adequate glucose uptake and metabolism are crucial for proper differentiation of the endometrium into a receptive state that supports embryo implantation (Frolova and Moley, 2011a). In addition, changes in epithelial cell structure and function in the receptive state, including increased adhesion molecules, downregulation of E-cadherin, and changes in polarity, are strongly associated with embryo attachment to the endometrial epithelial layer (Quinn et al., 2020). It is not hard to imagine that the physiological and metabolic changes of endometrial epithelial cells may depend on glucose metabolism. The ability to absorb and metabolize glucose at the cellular level is a characteristic shared by most living organisms. The same applies to the process of embryo implantation. The first step in glucose metabolism is uptake into the cell, which is performed by facilitative glucose transporters (GLUTs) which are membrane transport proteins in the GLUT family. Data on glucose utilization and metabolism are incomplete during implantation, both in terms of the development of invasive blastocysts and the establishment of endometrial receptivity.

Most cells import glucose by promoting diffusion mediated by GLUTs. Among the 14 identified transporters, fructose, inositol, and urate are substrates for transporters other than glucose. At least half of the major GLUT physiological substrates are uncertain or unknown (Thorens and Mueckler, 2010). The GLUT members are divided into three classes according to sequence similarity, and the GLUT proteins consist of about 500 amino acid residues, containing a single *N*-linked oligosaccharide site and 12 transmembrane domains (Mueckler and Thorens, 2013). Of the 14 family members, nine are expressed in the mouse uterus, and seven are expressed in the human uterus. GLUTs may be involved in establishing endometrial receptivity. Yamaguchi et al. (1996) reported that glucose transporter 1 (GLUT1) mRNA is initially detected in the decidua of rats and increases during pregnancy, suggesting that GLUT1 may play a role in maintaining pregnancy and fetal development. In addition, the “window of receptivity” (WOR) is the well-defined period during a female reproductive cycle when the endometrium is receptive to implanting an embryo, and the WOR of uterine physiology is primarily regulated at the level of gene expression by 17β-estradiol (E2) and progesterone (P4) through the estrogen receptor (ER) and progesterone receptor (PR), together with many transcription factors and co-regulators (Vasquez and DeMayo, 2013; Young, 2013). Recent studies have started to include these steroid hormones that regulate glucose metabolism through the expression of GLUTs (Frolova et al., 2009; Shi et al., 2014). However, the specific regulatory mechanism and the independent contribution of each hormone remain unclear.

In this study, we identified the effect of P4 on GLUT1 using an *in vivo* animal model, and determined the regulatory effect of P4 on glucose metabolism in endometrial epithelial cells through the detection of glycolysis representative products and key enzymes regulating the pentose phosphate pathway (PPP). In addition, the embryo implantation rate was significantly reduced after the GLUT1 small interfering RNA (siRNA) was injected into the uterine horn, and this suggests that GLUT1 may cause endometrial dysfunction by affecting glucose metabolism, thereby interfering with embryo implantation. We highly suspect that this pregnancy failure may be related to reduced GLUT1-mediated glucose metabolism, resulting in reduced endometrial receptivity due to an inadequate energy supply and substrate synthesis. Here, we propose a possible mechanism to explain how embryo implantation is affected by P4 and glucose utilization under abnormal endometrial conditions. Hopefully, our work will provide a little help in maintaining a woman’s pregnancy in the future.

## Materials and Methods

### Clinical Samples

Endometrial tissues (*n* = 30, 15 cases in the proliferative, and 15 cases in the secretory stage) were obtained from the First Affiliated Hospital of Dalian Medical University, China. These patients, aged 30–45 years, experienced non-endometrial lesions such as uterine fibroids, ovarian tumors, or cervical abnormalities, presented with a normal menstrual cycle, and did not experience hormonal disruption or receive hormonal medication. During the operation of obstetrics and gynecology, we obtained the consent of the patient, and showed respect and gratitude to the patient. The paraffin sample was confirmed by the pathologist and divided into mid, late-proliferative and mid, and late-secretory phase. This project was approved by the Ethics Committee of First Affiliated Hospital of Dalian Medical University, China. Written informed consent to participate in the study was obtained from the patient for use of their samples.

### Pregnancy Mouse Model

Wild-type C57 mice (8–10 weeks old, *n* = 5 in each group) were purchased from the Laboratory Animal Service Center of Dalian Medical University, China. Mice were fed in a room with a constant temperature (21°C) under a 12-h light/dark cycle. This study was approved by the Animal Ethics Committee of Dalian Medical University, China. Female mice in estrus were caged 1:1 with male mice. The vaginal plugs of the female mice were checked on the second day morning after mating, and that day was recorded as the first day (D1) after mating (Ruan et al., 2012; Sun et al., 2014). The D1–D5 mice uterine tissues were collected. Some of the tissues were stored at −80°C, and the remaining uterine tissues were fixed in 4% paraformaldehyde for immunohistochemistry (IHC).

### Intrauterine Injection

The mice (*n* = 5) were anesthetized with 0.2 ml 10% chloral hydrate on D3, skin incisions were made in the dorsal midline, and two small incisions were made in the muscle wall near the ovary to expose the uterine-oviduct connecting region. Mus-GLUT1 siRNA (80 pmol; sense, 5'-GCUUAUGGGCUUCUCCAAATT-3'; antisense, 5'-UUUGGAGAAGCCCAUAAGCTT-3'), or a scrambled siRNA negative control (NC; sense, 5'-UUCUCCGAACGUGUCACGUTT-3'; antisense, 5'-ACGUGACACGYYCGGAGAATT-3'; GenePharma, Shanghai, China) was mixed with lipofectamine 2000 (5 μl) and saline to 20 μl and injected into the lumen of each uterine horn at the uterine-oviduct connecting region (Bagot et al., 2000; Guo et al., 2014; Vallejo et al., 2018). The uterine tissue was collected on D7, and the number of implanted embryos was determined.

### Ovariectomized Mouse Model

A bilateral oophorectomy was performed on mice under anesthesia, and the mice were fed for 2 weeks to consume endogenous hormones. Then, the mice (*n* = 5 in each group) were intraperitoneally injected with either peanut oil (100 μl) or P4 (1 mg in a final volume of 100 μl; Sigma, St. Louis, MO, United States) for 4 consecutive days. Untreated mice were used as positive control. The mice were euthanized after 48 h, and the uterine tissue was collected. Some of the uterine tissues were stored at −80°C, and the remaining tissue was fixed in 4% paraformaldehyde for IHC.

### Cell Culture and Treatments

The RL95-2 (as a model of receptive endometrial epithelial cells) and JAR cell lines (as an embryo model; Kuramoto et al., 1972; Way et al., 1983; Hohn et al., 2000; Dominguez et al., 2003; Hannan et al., 2010) were obtained from the American type tissue culture collection (ATCC, Manassas, VA, United States). The JAR cells were grown in RPMI 1640 media (Thermo Fisher Scientific, Waltham, MA, United States) at 37°C with 5% CO_2_, respectively. The media were supplemented with 10% fetal bovine serum (FBS; Thermo Fisher Scientific, Waltham, MA, United States), 100 U/ml penicillin, and 100 mg/ml streptomycin. RL95-2 cells were grown in DMEM/F-12 medium supplemented with 0.005 mg/ml insulin and under the same culture conditions. Cells at an 80% density were treated with 0, 10, or 100 nM P4 (Sigma, St. Louis, MO, United States), the 0 nM was added consistent DMSO as control. Moreover, the cells were treated with 10 μM RU486 (Sigma, St. Louis, MO, United States), 100 nM P4, or RU486 and P4 and harvested for protein after a 48-h treatment. The cells were starved for 8 h prior to cell transfection, and transfected with the GLUT1 siRNA (100 pmol) using 5 μl lipofectamine 2000 (Invitrogen, Carlsbad, CA, United States) on each 35-mm plate. The homo-GLUT1 siRNA (sense, 5'-CUGUGGGCCUUUUCGUUAAtt-3'; antisense, 5'-UUAACGAAAAGGCCCACAGag-3'; Sawayama et al., 2019) and a scrambled sequence NC (sense, 5'-UUCUCCGAACGUGUCACGUTT-3'; antisense, 5'-ACGUGACACGYYCGGAGAATT-3') were purchased from GenePharma (Shanghai, China).

### *In vitro* Adhesion and Invasion Assay

RL95-2 cells were cultured in 35-mm plates (3 × 10^5^ cells) and grown with the transfected GLUT1 siRNA or NC treatments. JAR cells were pretreated with diluted (0.5 mM) CellTrace™ CFSE (Invitrogen, Carlsbad, CA, United States) medium for 30 min, and the cells suspension was added to plates containing RL95-2 cell monolayer, which were gently shaken (40 rpm) 1 h at 37°C (Choi et al., 2017; Xu et al., 2018). Then, the cell culture supernatant was removed, the plates were washed with PBS, the images were acquired using a fluorescent microscope, and the number of attached cells were counted using Image-Pro Plus software (Media Cybernetics Inc., Silver Spring, MD, United States). For invasion assay, briefly, RL95-2 cells (1 × 10^5^) were seeded into the 24-well Transwell, with Matrigel matrix (BD Biosciences, Franklin Lakes, NJ, United States). After incubation at 37°C for 24–48 h, the cells of invading to the bottom of the 8-μm pore filter membrane were stained with crystal violet and observed by a microscope. And the number of cells were counted using Image Pro-Plus software (Media Cybernetics Inc., Silver Spring, MD, United States).

### Glucose Uptake Assay

To measure glucose uptake, the RL95-2 cells were seeded in 24-well plates (8 × 10^4^ cells) and treated with P4 (0, 10, and 100 nM) for 48 h. The cells were incubated with a fluorescent D-glucose derivative, 2-[N-(7-nitrobenz-2-oxa-1,3-diazol-4-yl)amino]-2-deoxy-D-glucose (0.2 mM 2-NBDG; APExBIO Technology LLC., Houston, TX, United States) in glucose-free medium for 20 min at 37°C. Images were acquired using a fluorescent microscope, and glucose uptake rate was determined by Image Pro-Plus software and a fluorescein analysis.

### Pyruvate, Lactate, and ATP Assays

Intracellular pyruvate, lactate, and ATP levels were determined using the Micro Pyruvate Assay Kit (Solarbio, Beijing, China), CheKine™ Lactate Assay Kit (Abbkine, Wuhan, Hubei, China), and ATP detection Assay Kit (Solarbio, Beijing, China), respectively. After treating with P4 (0, 10, and 100 nM), the cells were lysed to measure intracellular pyruvate, lactate, and ATP levels following the manufacturer’s recommendations for each kit. Pyruvate and ATP levels were normalized to cell number, and lactate levels were normalized to total protein levels.

### Cellular Bioenergetics Analysis

The extracellular acidification rate (ECAR), as an indicator of glycolysis, was measured with a Seahorse Biosciences XF Analyzer (Seahorse Bioscience Inc., North Billerica, MA, United States) according to the manufacturer’s instructions. The cells after the various treatments were seeded in a Seahorse XF 24-well assay plate at a density 10,000 cells/well overnight for attachment. Then, the cells were washed, and the media were replaced with running media (DMEM of glucose-free and no phenol red, but supplemented with 10 mM glucose, 1 mM sodium pyruvate, and 2 mM glutamine, pH 7.4). The basal ECAR was determined for 30 min.

### Cell Proliferation Assay

Proliferation and viability of the cells were quantified with the Cell Counting Kit-8 (CCK-8) assay (Dojindo, Kojimindo, Japan). Briefly, after the P4 treatment, the cells were trypsinized and seeded in a 96-well plate (3,000 cells/well). Then, 10 μl of CCK-8 was added to each well, and the cells were incubated at 37°C for 2 h. Absorbance was measured at 450 nm using a Fluoroskan Ascent FL spectrophotometer (ThermoFisher Scientific, Waltham, MA, United States).

### Immunohistochemistry and Glycogen Analysis

The uterine endometrial tissue sections were deparaffinized in xylene. The sections were rehydrated in a descending concentration of ethanol, following antigen retrieval in citrate buffer (pH 6.0). After endogenous peroxidase activity was quenched with 0.3% H_2_O_2_, the sections were blocked in FBS at room temperature for 30 min. Then, specified antibodies to GLUT1 (Proteintech, Wuhan, China; 66290-1-Ig, 1,000 μg/ml) at a 1:100 dilution was applied overnight at 4°C, and the negative control performed the same experimental procedure, except that the primary antibody was replaced with PBS. Immunodetection was carried out by the diaminobenzidine-HRP reaction system (ZSGB-Bio Co., Beijing, China). For glycogen analysis, the sections were incubated with periodic acid Schiff (PAS) reagent (D004-1, Nanjing Jiancheng, Nanjing, China) according the manufacturer’s protocol and counter-stained with hematoxylin. Images were captured and analyzed using Image Pro-Plus software.

### Real-Time Polymerase Chain Reaction Analysis

Total RNA was isolated from the tissue samples using Trizol reagent (Takara, Shiga, Japan), and reverse transcribed into cDNA with the All-in-One First-Strand cDNA Synthesis SuperMix for qPCR assay kit (TransGen Biotech, Beijing, China) according to the manufacturer’s recommendations. Quantitative PCR was performed using the Top Green qPCR SuperMix Kit (TransGen Biotech, Beijing, China) on a step-one real-time PCR System (Applied Biosystems, Foster City, CA, United States). The reaction conditions were 95°C for 5 min, 95°C for 15 s and 35 cycles, 60°C for 60 s, and 72°C for 5 min. The primer sequences were: homo-GLUT1: forward, 5'-CCAGCTGCCATTGCCGTT-3'; reverse, 5'-GACGTAGGGACCACACAGTTGC-3', GLUT3: forward, 5'-CAATGCTCCTGAGAAGATCATAA-3'; reverse, 5'-AAAGCGGTTGACGAAGAGT-3', Homo-β-actin: forward, 5'-TGTTTGAGACCTTCAACACC-3'; reverse, 5'-ACGCAGGATGGCATGG-3'; Mus-Glut1: forward, 5'-AGCCCTGCTACAGTGTAT-3', reverse, 5'-AGGTCTCGGGTCACATC-3'; Mus-Hoxa10: forward, 5'-CCTGCCGCGAACTCCTTTT-3', reverse, 5'-GGCGCTTCATTACGCTTGC-3'; Mus-Gapdh: forward, 5'-AGACAGCCGCATCTTCTTGT-3', reverse, 5'-ATCCGTTCACACCGACCTTC-3'.

### Western Blotting Analysis

The cell proteins were extracted with radioimmunoprecipitation assay lysis buffer (KeyGen BioTECH, Beijing, China) with protease and phosphatase inhibitors. The proteins (40 μg) were subjected to 10% sodium dodecyl sulfate-polyacrylamide gel electrophoresis and transferred to a nitrocellulose filter membrane (Millipore, Billerica, MA, United States). The membranes were blocked with 5% skim milk at room temperature for 2 h and incubated with the GLUT1 (1:1,000; Proteintech, Wuhan, China; 66290-1-Ig, 1,000 μg/ml), glucose-6-phosphate dehydrogenase (G6PD) (1:5,000; Proteintech, Wuhan, China; 66373-1-Ig, 1,000 μg/ml), and β-actin (1:1,000; Proteintech, Wuhan, China; 20536-1-AP, 430 μg/ml) antibodies overnight at 4°C. The signal was detected with HRP-conjugated secondary antibody (1:3,000; Proteintech, Wuhan, China) for 1 h at room temperature and visualized by enhanced chemiluminescence detection reagents (ThermoFisher Scientific, Waltham, MA, United States). Densitometry analysis was performed by Image Lab 4.0 software (Bio-Rad Laboratories, Hercules, CA, United States).

### Statistical Analysis

All data are presented as mean ± SD. Differences between groups were detected using Student’s *t*-test using GraphPad Prism 8.0 (GraphPad Software, San Diego, CA, United States). A value of *p* < 0.05 was considered significant. All experiments were independently performed at least three times.

## Results

### Endometrial GLUT1 Expression Is Upregulated During the Peri-Implantation Period

**Figure 1:**
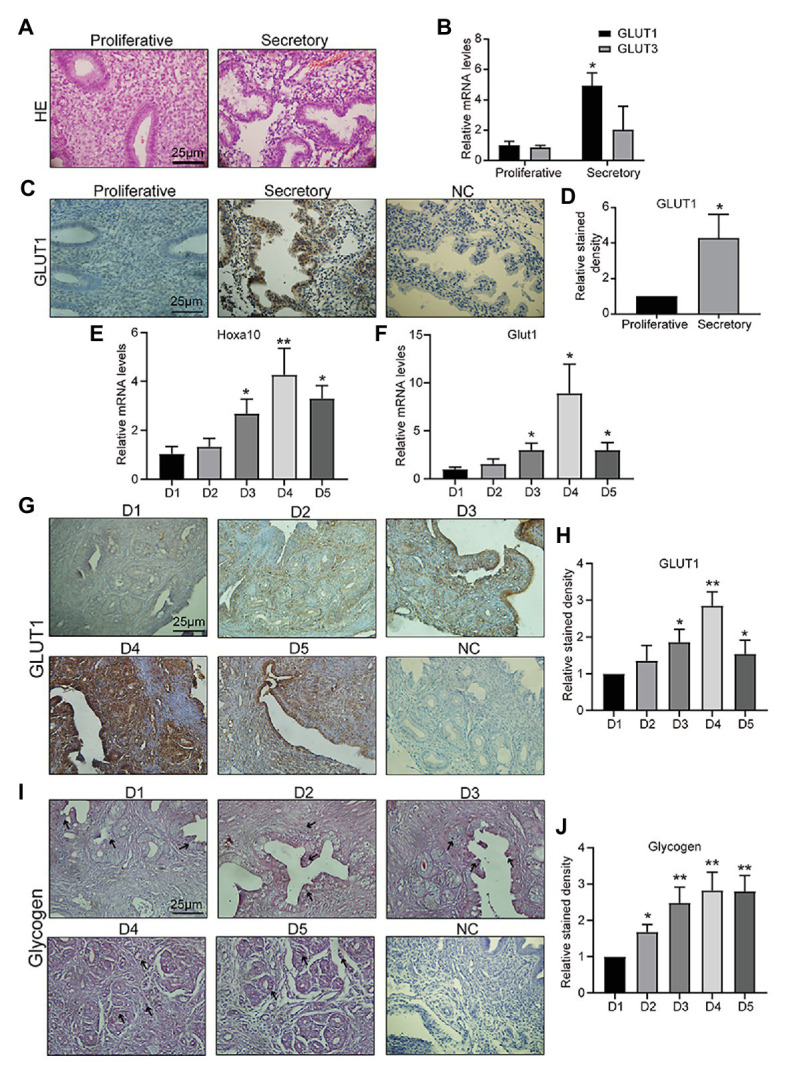
Glucose transporter 1 (GLUT1) expression during the peri-implantation period. **(A)** HE staining of human endometrial tissue (*n* = 5, scale bar = 25 μm). **(B)** The quantitative PCR (qPCR) analysis of GLUT1 and GLUT3 mRNA levels in human endometrial tissues at different phases (*n* = 10, normalized by β-actin, ^*^ vs. proliferative). **(C)** Immunohistochemistry (IHC) staining of the GLUT1 in the human endometrial tissues at different phases (*n* = 10, scale bar = 25 μm). **(D)** Relative stained density of GLUT1 in human endometrial tissue was counted with Image Pro-Plus software. ^*^ vs. Proliferative. **(E)** The qPCR analysis of Hoxa10 mRNA levels in mice uterus tissues on D1–D5 (*n* = 5, normalized by Gapdh, ^*^ vs. D1). **(F)** The qPCR analysis of GLUT1 mRNA levels in mice uterus tissues on D1–D5 (*n* = 5, normalized by Gapdh, ^*^ vs. D1). **(G)** IHC staining of GLUT1 protein in the mice uterus tissues on D1–D5 (*n* = 5, scale bar = 25 μm). **(H)** Relative stained density of GLUT1 in mice uterus tissues was counted with Image Pro-Plus software. ^*^ vs. D1. **(I)** The glycogen in the mice uterus tissues of D1–D5 were analyzed by periodic acid Schiff (PAS) staining assay kit (*n* = 5; scale bar = 25 μm). **(J)** Relative stained density of glycogen in mice uterus tissues was counted with Image Pro-Plus software. ^*^ vs. D1. Error bars represent the mean ± SD. Student’s *t*-test, ^*^ *p* < 0.05, ^**^ *p* < 0.01.

Appropriate endometrial glucose metabolism is important for endometrial differentiation and decidualization, and for providing nutrition and a receptive environment for the embryo. The first step in glucose metabolism is uptake of glucose into cells, which involves diffusion mediated by facilitative GLUTs in most mammalian cells. GLUT1–5 were the first family members to be described, and therefore have been the most studied. Specifically, GLUT2 has been reported not to be expressed in mouse or human endometrium by multiple groups (von Wolff et al., 2003; Frolova and Moley, 2011b). GLUT5 is not a glucose transporter, but a fructose transporter, which is abundantly expressed in sperm (Angulo et al., 1998). GLUT5 is also not detected in the human endometrium (Frolova and Moley, 2011b). GLUT4 is the important insulin-sensitive transporter (Jagadish et al., 1996). In this study, firstly, clinical samples of endometrial tissue were collected, and the pathologist diagnosed them as mid, late-proliferative and mid, and late-secretory phase by HE staining (Figure 1A). Then, we detected *GLUT1* and *GLUT3* mRNAs in human endometrium tissue and found that the *GLUT1* expression level in human endometrium was higher during the secretory phase than during the proliferative phase, but no difference was observed in *GLUT3* (Figure 1B). We then examined GLUT1 expression in human endometrium by IHC. The results showed that the GLUT1 expression was consistent with mRNA level in the human endometrium (Figures 1C,D), and that its localization is mainly in glandular epithelium. Hoxa10 is one of the most promising candidates for measuring endometrial receptivity and is involved in implantation and decidualization (Daikoku et al., 2005; Godbole et al., 2007; Modi and Godbole, 2009). Furthermore, we developed pregnant mouse models and validated D1–D5 uteri through Hoxa10 (Figure 1E). Glut1 mRNA level was assessed during D1–D5 in the pregnant mouse models, and the results showed a significant increase in D4 and D5 to D1–2 (Figure 1F). And the expression of GLUT1 is consistent with the results of IHC (Figures 1G,H). In addition, we find that GLUT1 massively increases in stromal cells of D4, indicating a drastic energy change, which is indirectly verified by the accumulation of glycogen (Figures 1I,J). And in this process, P4 begins to predominate in humans and mice. This finding suggests that GLUT1 may be induced by P4.

### Endometrial GLUT1 Expression Is Induced by Progesterone

**Figure 2:**
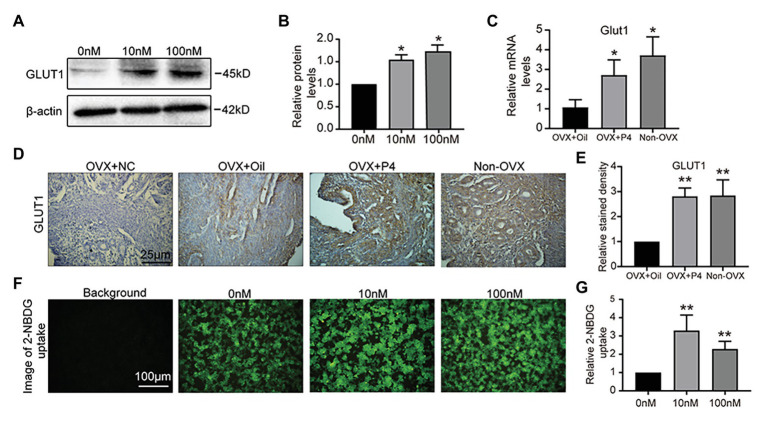
GLUT1 expression is induced by progesterone (P4). **(A)** Western blot analysis of GLUT1 in RL95-2 cells treated with P4 (0, 10, and 100 nM, 0 nM group was added consistent DMSO as control) for 48 h. **(B)** Relative density analysis of the GLUT1 protein by Image Lab 4.0 software (*n* = 3, ^*^ vs. 0 nM). **(C)** The qPCR analysis of GLUT1 mRNA levels in the ovariectomized (OVX) mouse uterus treated with oil or P4 (*n* = 5, normalized by Gapdh, ^*^ vs. OVX + oil, non-OVX as the positive control). **(D)** IHC staining of the GLUT1 in the uterus of OVX + oil, OVX + P4, and non-OVX mice (*n* = 5, scale bar = 25 μm). **(E)** Relative stained density of GLUT1 in mice uterus tissues was counted with Image Pro-Plus. ^*^ vs. OVX + oil, non-OVX as the positive control. **(F)** Fluorescent image of 2-[N-(7-nitrobenz-2-oxa-1,3-diazol-4-yl)amino]-2-deoxy-D-glucose (2-NBDG) uptake in cells treated with P4 (0, 10, and 100 nM) for 48 h. **(G)** Relative 2-NBDG uptake ratio was analyzed by Image Pro-Plus (*n* = 3). Error bars represent the mean ± SD. Student’s *t*-test, ^*^ *p* < 0.05, ^**^ *p* < 0.01.

We hypothesized that GLUT1 may be directly regulated by P4 *in vivo*. So, we treated the epithelial cell model (Kuramoto et al., 1972; Way et al., 1983; Hohn et al., 2000; Dominguez et al., 2003; Hannan et al., 2010) with P4, and found that P4 increased the expression of GLUT1 (Figures 2A,B). Furthermore, we prepared an ovariectomized (OVX) mouse model to reveal the effects of P4 and found that P4 was responsible for upregulating the GLUT1 protein in the murine uterus compared with the oil group (Figures 2C-E). Endometrial cell proliferation, stromal cell decidualization, and differentiation at the time of implantation and during early pregnancy depend on adequate glucose metabolism (Frolova and Moley, 2011a). Interestingly, the P4 treatments promoted glucose uptake in the cell model (Figures 2F,G). Although enhanced glucose influx is critical for endometrial proliferation and decidualization, the mechanism regulating glucose metabolism is not fully understood. We were interested in determining whether P4 could provide energy for the endometrium during implantation and early pregnancy by increasing glycolytic metabolism.

### Progesterone Affects Glucose Metabolism and Cell Function

**Figure 3:**
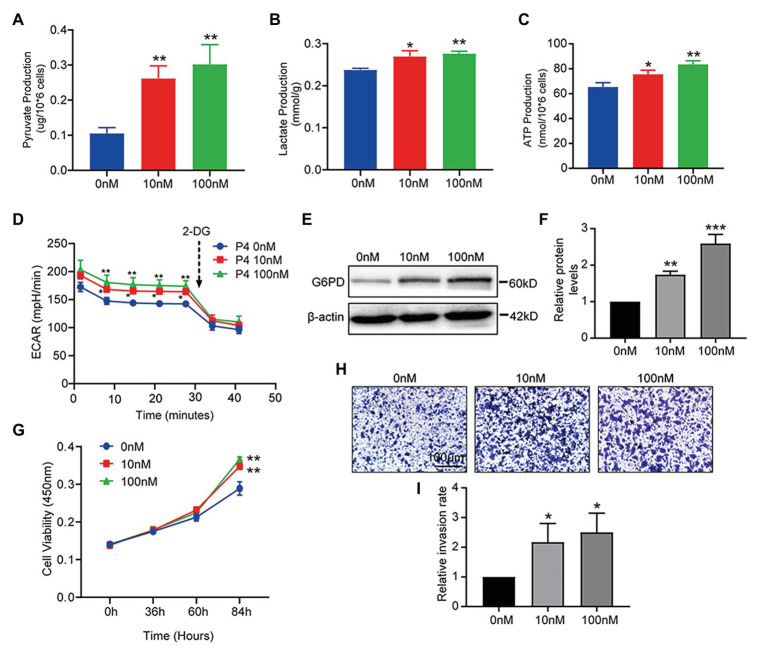
P4 activates glucose metabolism and endometrial cell proliferation. RL95-2 intracellular pyruvate **(A)**, lactate **(B)**, and ATP levels **(C)** were detected using assay kits after stimulation with P4 (0, 10, and 100 nM) for 48 h (*n* = 3, ^*^ vs. 0 nM). **(D)** The extracellular acidification rate (ECAR; mpH/min) was measured under basal conditions in RL95-2 cells treated with different P4 concentrations (0, 10, and 100 nM). **(E)** Western blot analysis of glucose-6-phosphate dehydrogenase (G6PD) in RL95-2 cells treated with P4 (0, 10, and 100 nM) for 48 h. **(F)** Relative density analysis of the G6PD protein Image Lab 4.0 software (*n* = 3, ^*^ vs. 0 nM). **(G)** Cell proliferation assays of RL95-2 cells treated with P4 (0, 10, and 100 nM) for 48 h by Cell Counting Kit-8 (CCK-8). **(H)** Cell invasion in P4 (0, 10, and 100 nM)-treated RL95-2 cells by Transwell assay (scale bar = 100 μm). **(I)** Relative invasion rates were analyzed by Image Pro-Plus software (*n* = 3). Error bars represent the mean ± SD. Student’s *t*-test, ^*^ *p* < 0.05, ^**^ *p* < 0.01, ^***^ *p* < 0.001.

The results above show that P4 increased glucose uptake at the cellular level. To further clarify whether P4 activates glucose uptake and glycolysis, we measured three indicators of glycolysis, such as intracellular pyruvate, lactate, and ATP levels at different P4 concentrations. We observed a significant simulative effect of P4 on the three indicators of glycolysis in the cell model after a 48-h treatment with several P4 concentrations (Figures 3A–C). To further reflect the glycolytic level in real-time, the ECAR as an approximation of glycolysis was determined using the Seahorse flux analyzer. The result showed that 10, 100 nM P4 caused a significant increase in basal glycolysis (Figure 3D). To elucidate the glucose utilization pathway, we analyzed G6PD expression in the PPP, and it was increased after the P4 treatment (Figures 3E,F). This result indicates that the PPP was enhanced by P4. In addition, the ability of these cells to grow and proliferate was also affected by P4 (Figure 3G). Meanwhile, we found that P4 also affects the ability of cells to invade (Figures 3H,I). This suggests that P4 may induce glucose uptake by increasing GLUT1 expression. In addition, glucose uptake is utilized for glycolysis and PPP to provide energy and a synthetic substrate for cellular metabolic and functional changes.

### The Absence of GLUT1 Inhibits Glycolysis Levels

**Figure 4:**
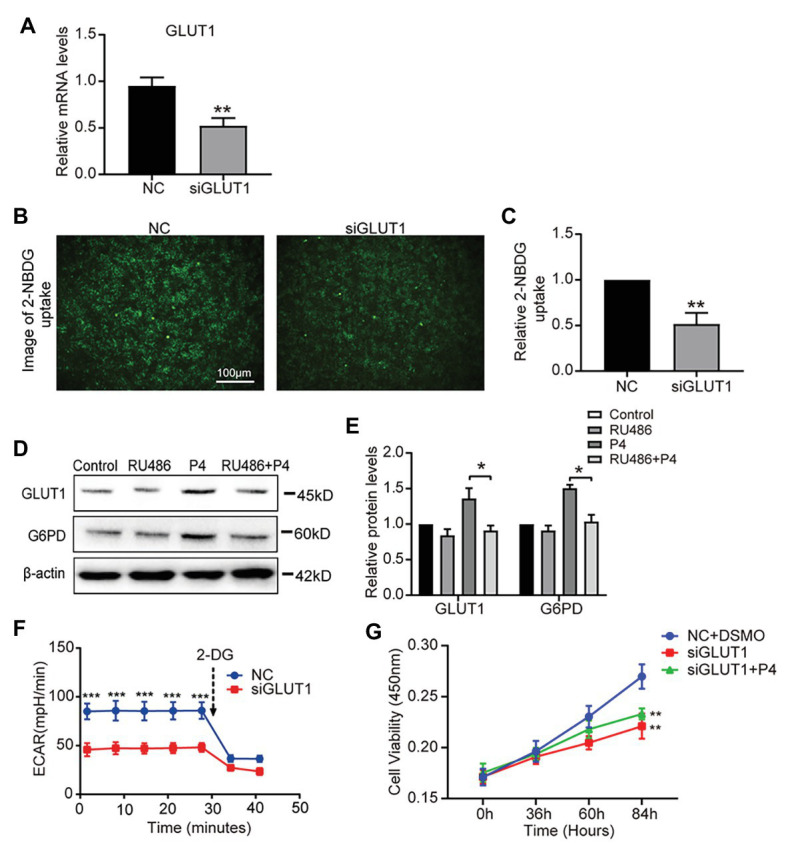
The down-regulation of GLUT1 inhibits glycolysis and proliferation. **(A)** The qPCR analysis of GLUT1 mRNA levels in RL95-2 cells treated with negative control (NC) small interfering RNA (siRNA) or GLUT1 siRNA (*n* = 3, normalized by β-actin). **(B)** Fluorescent image of 2-NBDG uptake into cells treated with NC siRNA or GLUT1 siRNA (scale bar = 100 μm) **(C)** The relative 2-NBDG uptake ratio was analyzed by Image-Pro Plus software. **(D)** Western blot analysis of GLUT1 and G6PD in RL95-2 cells treated with control, P4 (10 nM), RU486 (10 μM), and P4 + RU486. The cells were pretreated with RU486 for 1 h before the P4 treatment. **(E)** Relative density analysis of the GLUT1 and G6PD proteins by Image Lab 4.0 software (*n* = 3). **(F)** The ECAR (mpH/min) was measured under basal conditions in RL95-2 cells treated with NC siRNA or GLUT1 siRNA (*n* = 3). **(G)** Cell proliferation assays of RL95-2 cells treated with NC + DSMO, siGLUT1, and siGLUT1 + P4 (100 nM) by CCK-8; *n* = 3, ^*^ vs. NC + DSMO. Error bars represent the mean ± SD. Student’s *t*-test, ^*^ *p* < 0.05, ^**^ *p* < 0.01, ^***^ *p* < 0.001.

To determine the effect of P4 on glycolysis caused by GLUT1, we downregulated GLUT1 by small interfering RNA (RNAi) and verified the effect of interference (Figure 4A), and found that the reduction of GLUT1 could inhibit glucose uptake (Figures 4B,C). To further test this hypothesis, we treated the cells with the progesterone receptor antagonist RU486 and determined the expression of GLUT1 and G6PD. The WB results showed that increased levels of P4-mediated GLUT1 and G6PD were inhibited by the RU486 (Figures 4D,E). In addition, downregulating GLUT1 can lower the ECAR level (Figure 4F). Furthermore, we detected the proliferation of cells under the 100 nM P4 after knockdown GLUT1 by siRNA. Results showed that P4 does not promote cell proliferation when GLUT1 is low (Figure 4G). Thus, the P4-induced increase in glucose metabolism depends on GLUT1.

### Knockdown of GLUT1 Results in Failed Embryo Implantation *in vivo*

**Figure 5:**
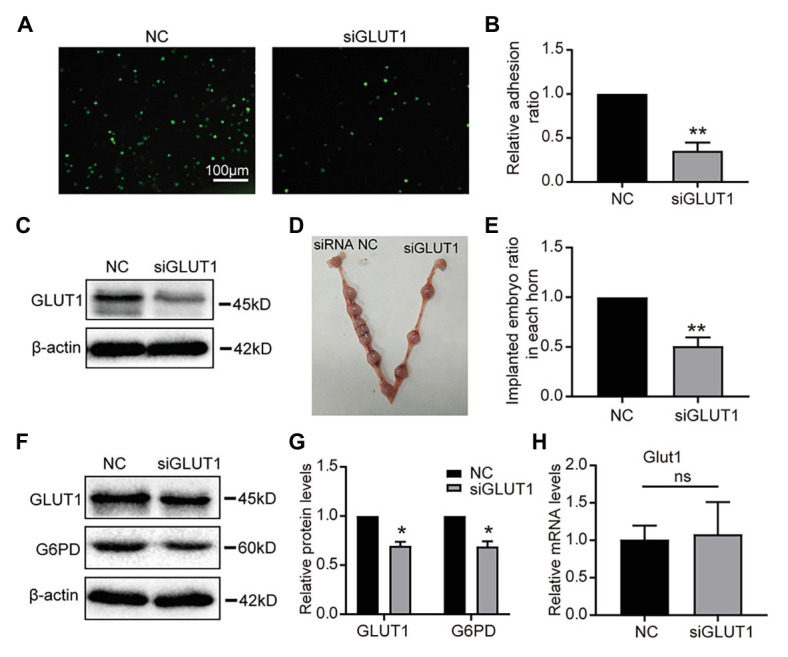
Effect of GLUT1 on embryo implantation in mice. **(A)** Adhesion of JAR cells onto RL95-2 endometrial epithelial cells was observed in response to GLUT1 silencing *in vitro* (scale bar = 100 μm). **(B)** The relative adhesion ratio of JAR cells on endometrial cells was analyzed by Image-Pro Plus software (*n* = 3). **(C)** Western blot analysis of GLUT1 in RL95-2 cells treated with NC siRNA or GLUT1 siRNA. **(D)** Typical photograph showing mice uteri on D7 of mating after injecting mus-NC siRNA (left) or GLUT1 siRNA (right). **(E)** The implanted embryos ratio was analyzed after injecting NC siRNA or GLUT1 siRNA (*n* = 5). **(F)** Western blot analysis of GLUT1 and G6PD in mice uteri on D7 of mating after injecting mus-NC siRNA (left) or GLUT1 siRNA (right). **(G)** Relative density analysis of the GLUT1 and G6PD by Image Lab 4.0 software (*n* = 3). **(H)** The qPCR analysis of Glut1 mRNA levels of embryos in the uterus of the transfection group and the NC group (*n* = 3, normalized by Gapdh, ns, no significant difference). Error bars represent the mean ± SD. Student’s *t*-test, ^*^ *p* < 0.05, ^**^ *p* < 0.01.

To assess the role of GLUT1 in embryo implantation, we first observed that the Silencing GLUT1 significantly reduced the number of trophoblast cells attached to endometrial cells compared to the control group in a commonly used *in vitro* adhesion model (Figures 5A,B), and the silence effect of GLUT1 was verified by WB (Figure 5C). Next, we injected GLUT1 siRNA into the uterine horn of pregnant mice on D3 and injected NC scrambled siRNA into the other side as a control. The results showed that significantly fewer embryo implantations occurred after GLUT1 siRNA was injected than that of the control group (Figures 5D,E). At the same time, the GLUT1 and G6PD expression levels decreased significantly in the uteri tissues treated with GLUT1 siRNA compared with the control (Figures 5F,G). In addition, we detected GLUT1 mRNA of intrauterine embryos in the transfection group and found no difference (Figure 5H). This is also consistent with previous studies (Bagot et al., 2000), suggesting that GLUT1 silencing affects the endometrium rather than directly affecting the embryo. This finding suggests that GLUT1 is required for successful implantation.

## Discussion

Successful embryo implantation requires an intimate interaction between an invasive embryo and a receptive endometrium. Appropriate differentiation of the endometrium during a critical period is required for this interaction during the implantation window. This process is mainly mediated by P4 and to some extent by E2 (Frolova et al., 2009). P4 is essential for embryo implantation and pregnancy maintenance, the notion that infertility is caused by a lack of P4 is logically irrefutable. As early as 1934, Zuckerman and Fulton (1934) demonstrated the effects of P4 on the endometrium in non-human primates. In 1973, Jones (1973) described P4 deficiency in patients who may contribute to infertility. This allowed the important role of P4 to be slowly revealed. The data from team of Usadi et al. (2008) strongly suggest that a wide range of P4 concentrations can affect changes in normal secretory phase endometrial structure and function in young healthy women. However, it is not clear whether P4, as an initial signal, is involved in the glucose metabolism of endometrium, which makes us have a strong hunch that P4 may play an important role in the glucose metabolism of normal secretory phase endometrium.

A blastocyst entering the uterine cavity must first attach to the epithelial cells, and then invade the underlying stromal cells that differentiate into the decidua. At this point, functional defects of epithelial cells may directly affect embryo recognition and adhesion. The blastocyst is implanted only when direct cross-talk between the embryo and the maternal endometrium is established (Daikoku et al., 2011). Studies have shown that the expression of adhesion molecules in endometrial epithelial cells increases at the receptive stage (Fujiwara et al., 2020), most of which are transmembrane glycosylated modified proteins, while the glycosylated precursors, such as UDP-GlcNAc and UDP-GalNAc, are mostly derived from the branches of glucose metabolism. This suggests the role of glucose metabolism in regulating epithelial cell function. In addition, after embryo attachment, intraepithelial invasion begins, and the epithelial cell layer is opened. Epithelial-mesenchymal transformation (EMT) has been shown to be involved in the opening of the epithelial layer (Uchida et al., 2012; Owusu-Akyaw et al., 2019). Our previous studies have shown that P4 can mediate ezrin remodeling and cytoskeletal rearrangement through aquaporin-3, affecting epithelial cell motility and participating in EMT (Cui et al., 2018). And the movement of epithelial cells is inevitably dependent on the supply of energy, which indirectly indicates that the regulation of P4 on cells is involved in glucose metabolism.

It has been reported that GLUT1 mRNA is the most abundant GLUT transcript in human and mouse endometrial stroma. Specifically, the reduction of GLUT1 expression leads to the reduced efficiency of endometrial decidualization (Frolova and Moley, 2011b). In this study, we observed that the expression of GLUT1 in the D4 stroma of mice was significantly increased, suggesting that the drastic metabolic changes during implantation might be a preparation for decidua. And this regulatory effect has been proven to involve P4 (Frolova et al., 2009). However, the specific regulatory mechanism of P4 on GLUT1 in endometrium is not clear. Further, it will be interesting to see whether the mechanisms by which P4 regulates epithelial and stromal cells are consistent, and what are the coordination links between them. In addition, changes in glucose concentration in the serum of pregnant women or the downstream metabolism of glucose can lead to failed implantation and subsequent miscarriage. In fact, studies have shown that GLUT1 expression decreases significantly in women with idiopathic infertility when compared with women who are infertile due to tubal occlusion or male factors (non-endometrial infertility; von Wolff et al., 2003). Therefore, idiopathic infertility in some patients may be due to a decrease in GLUT1 expression leading to decreased glucose uptake by endometrial cells and cellular dysfunction. It can be seen that glucose transporter-mediated glucose metabolism plays an important role in pregnancy outcomes during the establishment of endometrial cell receptive state and decidualization.

In this study, we defined GLUT1 expression in the human and mouse endometrium during the reproductive cycle. We determined whether P4 was a part of the upstream regulatory mechanism of GLUT1, given the expression of GLUT1 during peri-implantation. A recent study showed that steroid hormones play an important role in glucose utilization by altering GLUT expression in human breast cancer cell lines (Medina et al., 2003). However, the situation *in vivo* remains unclear. Next, we determined the effect of P4 on GLUT1 in an ovariectomized mouse model. Further *in vitro* studies indicated that upregulation of GLUT1 was mediated by P4 through the PR and reversed by the antagonist RU486. To determine the functional significance of the increased expression of GLUT1 protein, we measured glucose uptake capacity of the cells and subsequent glycolysis and PPP activities. The results confirmed that increased expression of GLUT1 enhanced the downstream metabolic pathway and may function as a major glucose transporter. These data are consistent with the results of Frolova et al. (2009) who reported *in vitro* regulation of GLUT1 by P4 in isolated mouse endometrial stromal cells. Interestingly, however, Frolova et al. (2009) also found that E2 reverses this effect of P4, bringing GLUT1 back to its base level in stromal cells. This finding is important in demonstrating the opposite effect of hormones on GLUT1. However, E2 and P4 are both present at the time of implantation, suggesting that they interact to regulate GLUT1 expression. In addition, after injecting siGLUT1 into the uterine horn of mice, the pregnancy rate of mice decreased significantly, suggesting that GLUT1 play an important role in embryo implanted WOR. But, the underlying mechanism of the endometrium from a pre-receptive to a receptive condition is unclear.

Our evidence confirms the effect of P4 on glucose metabolism through GLUT1; however, how the activation of P4 drives G6PD and PPP remains to be further studied. Meanwhile, the specific regulatory mechanism between P4 and GLUT1 is not clear. This provides a direction for our next work. However, the current development of omics provides us with good technical support, so that we can well determine the correlation between the two.

## Conclusion

**Figure 6:**
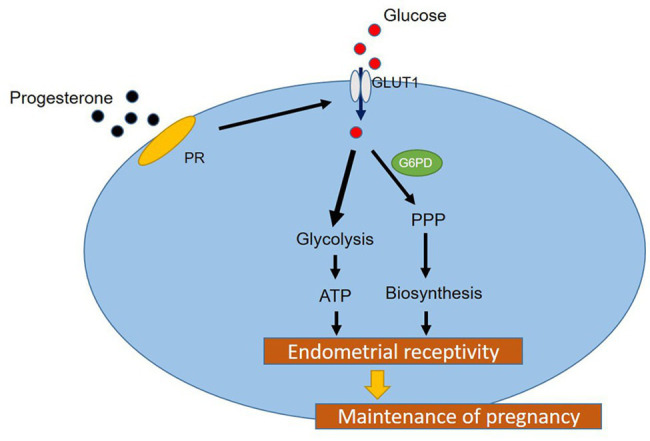
A hypothetical working model of the molecular regulatory mechanism of P4 on GLUT1 in glucose metabolism.

In conclusion, in this study, we identified the regulatory effect of P4 on GLUT1 through *in vivo* animal models, and determined the regulatory effect of P4 on cell glucose metabolism. And we highly suspect that pregnancy failure may be due to reduced GLUT1-mediated glucose metabolism, resulting in reduced endometrial receptivity and decidualization due to inadequate energy supply and substrate synthesis. Here, we propose a possible mechanism to explain how embryo implantation is affected by P4 and glucose utilization under abnormal endometrial conditions (Figure 6). Hopefully, our work will provide a little help in maintaining a woman’s pregnancy in the future.

## Data Availability Statement

All datasets generated for this study are included in the article/supplementary material.

## Ethics Statement

The studies involving human participants were reviewed and approved by Ethics Committee of First Affiliated Hospital of Dalian Medical University. The patients/participants provided their written informed consent to participate in this study. The animal study was reviewed and approved by the Animal Ethics Committee of Dalian Medical University.

## Author Contributions

YK, LK, and HZ conceived and designed the study. HZ wrote the manuscript. JQ and YW performed the main experiments. JS, ZL, and CL made the animal models. YS collected and provided the clinical samples. LS and JF reviewed the manuscript. All authors contributed to the article and approved the submitted version.

### Conflict of Interest

The authors declare that the research was conducted in the absence of any commercial or financial relationships that could be construed as a potential conflict of interest.
